# Comparative Pharmacokinetics and Preliminary Pharmacodynamics Evaluation of Piscidin 1 Against PRV and PEDV in Rats

**DOI:** 10.3389/fchem.2018.00244

**Published:** 2018-06-25

**Authors:** Zhixin Lei, Qianying Liu, Qianqian Zhu, Bing Yang, Haseeb Khaliq, Ao Sun, Yi Qi, Gopi Krishna Moku, Yafan Su, Jiawei Wang, Jiyue Cao, Qigai He

**Affiliations:** ^1^State Key Laboratory of Agriculture Microbiology, College of Veterinary Medicine, Huazhong Agriculture University, Wuhan, China; ^2^Department of Veterinary Pharmacology, College of Veterinary Medicine, Huazhong Agricultural University, Wuhan, China; ^3^National Reference Laboratory of Veterinary Drug Residues and MAO Key Laboratory for Detection of Veterinary Drug Residues, Huazhong Agriculture University, Wuhan, China; ^4^Department of Pharmaceutics, University of Minnesota, Minneapolis, MN, United States

**Keywords:** Piscidin-1, pharmacokinetics, bioavailability, ileum content, PEDV

## Abstract

Antimicrobial peptide (Piscidin-1) is an effective natural polypeptide, which has great influence and potential on porcine epidemic diarrhea virus (PEDV) and pseudorabies virus (PRV). As an alternative antibiotic substitute, Piscidin-1 was subjected for pharmacokinetics study with three administration routes (i.v, i.m, and p.o) after a single dose of 2 mg/kg in rats and preliminary pharmacodynamics including antiviral activity in cell against PEDV and PRV. Based on 50 percent tissue culture infective dose (TCID_50_), there were about 2 and 10% virus survived ratios for Piscidin-1 against PRV and PEDV, respectively. The plaque test showed 1 and 2 μg/ml Piscidin-1 could eliminate 95% PRV and 85% PEDV, respectively. The main pharmacokinetics parameters of C_max_, AUC_0−∞_, Ke, t_1/2_, T_max_, MRT, and Cl_b_ in plasma were not applicable value, 25.9 μg^*^h/ml, 0.041 h^−1^, 16.97 h, not available value, 22.77 h, 0.067 L/h^*^kg after i.v administration, 2.37 μg/ml, 18.95 μg^*^h/ml, 0.029 h^−1^, 23.50 h, 0.33 h, 30.12 h, 0.095 L/h^*^kg after i.m administration and 0.73 μg/ml, 9.63 μg^*^h/ml, 0.036 h^−1^, 19.46 h, 0.50 h, 26.76 h, 0.171 L/h^*^kg after p.o administration. The bioavailability values after i.m and p.o administrations were calculated as 73.17 and 37.18%, respectively. The i.m administration was selected for pharmacokinetics study in ileum content against PEDV. The main pharmacokinetic parameters of C_max_, AUC_0−∞_, Ke, t_1/2_, T_max_, MRT, and Cl_b_ in ileum content were 1.67 μg/ml, 78.40 μg^*^h/ml, 0.034 h^−1^, 20.16 h, 8.12 h, 36.45 h, 0.026 L/h^*^kg. The C_max_ values in plasma (2.37 μg/ml) and ileum content (1.67 μg/ml) were higher than the effective inhibitory concentration determined in the plaque test, and this indicates that Piscidin-1 might have effective inhibition effect against PRV and PEDV after administration of 2 mg/kg i.m. The results of this study represent the first investigations toward the pharmacokinetic characteristics of piscidin-1 in plasma upon three different administration routes, among which i.m. resulted in the highest bioavailability (73.17%). Furthermore, the pharmacokinetics study of ileum content indicated Piscidin-1 might have good effect against PEDV and could be regarded as an alternative antibiotic in clinical veterinary in the future study.

## Introduction

With the rapid development of bacterial resistance, the new antibiotics exploration is entering researchers' vision. As a potential candidate, antimicrobial peptides (AMPS) have aroused a high clinical interest studied by more and more researchers (Bell, 2003). As a new type of therapeutic agent, AMPS not only develops for its antibacterial action, but also shows the innate and adaptive immune response to the viruses at all stages of life (Rahmanpour et al., 2013; Lee et al., 2014; Kumar et al., 2017). Moreover, AMPs are also considered and developed as novel potential agents instead of antibiotics against Gram-negative bacteria, Gram-positive bacteria, viruses and even cancerous cells in the future.

Piscidin 1 belong to the piscidin family, is a type of AMPs produced by fish mast cells. Piscidin is regarded as a family of at least 3 proteins, 22-amino acid peptides and has a highly conserved, histidine and phenylalanine-rich N-terminus and a variable C-terminus (Silphaduang and Noga, 2001; Noga and Silphaduang, 2003). Piscidin 1 has shown the highest antibacterial activity and plays an important role in the innate immune system in the piscidin family (Silphaduang and Noga, 2001; Lauth et al., 2002; Noga and Silphaduang, 2003). Furthermore, as owning a LPS-neutralizing property, Piscidin 1 has potent activity against a variety of microbes, including filamentous fungi, Gram-positive and negative bacteria, and other resistant bacteria that have been reported in the previous researches (Lee et al., 2014; Kumar et al., 2016). Piscidin 1 has been reported to have the best antimicrobial activity of all piscidin family members against pathogenic bacteria such as *Escherichia coli, Pseudomonas aeruginosa, B. subtilis, S. epidermidis*, and *Staphylococcus aureus* with a low minimum inhibitory concentration (MIC) (≤ 5.14 μg/ml), and most of the previously published reports focus on the activity and mechanism against pathogens (Lee et al., 2014). However, it would focus on the pharmacologic action against viruses (PEDV and PRV) and *in vivo* pharmacokinetics (PK) profiles in pigs in this study.

PK evaluation plays a key role in new animal drugs development. Moreover, it could support a supplemental application for modification of administration routes, dosage forms, scheme, and manufacturing process which may make a significant effect for usage (Lei et al., 2017b,c, 2018; Zaid et al., 2017). As a newly developed antibiotic substitute, Piscidin 1 has barely been investigated for PK with different administrations and dosages. In addition, it is more and more popular to investigate the antibiotic concentrations in the target site in the animal.

Porcine epidemic diarrhea virus (PEDV) is characterized by acute enteric infection and high mortality in sucking pigs and could cause enormous economic losses to the swine industry (Li et al., 2012; Crawford et al., 2016). PEDV is also the serious virus disease, infecting intestinal tract in pigs (Islam et al., 2016; Guo et al., 2017). Few researchers have measured the drug concentrations at the target tissues in pigs. For intestinal tract infection, the main target tissue may be the ileum content. Since the difficulty in measuring the antibiotic concentrations at the infected site, the PK data were mostly determined from the serum of the animals in the most previous reports. However, the drug concentrations in plasma are significantly different to target sites which have been reported in the previously published reports such as the marbofloxacin and cyadox concentrations in ileum and plasma (Lei et al., 2017a,b,d, 2018; Yan et al., 2017). Thus, as a kind of intestinal tract infection virus, it is essential to investigate the PK profiles with an appropriate route in ileum content for Piscidin 1 against PEDV.

To our knowledge, there are rarely previous studies integrated the data of pharmacodynamics (PD) and PK for Piscidin 1 against PEDV and PRV with three modes of administration (i.m, i.v, and p.o). The aim of this study is to evaluate the comparative pharmacokinetics and preliminary pharmacodynamics of Piscidin 1. Because of the limit for the production of Piscidin 1, the research is carried out in rats as a primary animal model in this study.

## Materials and methods

### Cell, viruses, and chemicals

The PEDV and PRV variant strains (YN and YA) were isolated from sucking piglets and provided by state key laboratory of agricultural microbiology, college of veterinary medicine, Huazhong Agricultural University. The standard Piscidin 1 (powder) and lactoferricin (powder) are compounded by Wuhan New Oriental Biotechnology co. LTD. The amino acid sequence of Piscidin 1 and lactoferricin are as follow FFHHI FRGIV HVGKT IHRLV TG and FKCRRWQWRM KKLGAPSITC VRRAF. All the chemical reagents and organic solvents used were of HPLC grade. African green monkey kidney cell lines (Vero-E6 cell) and porcine kidney cell (PK-15 cell), were also provided by this lab and cultured in Dulbecco's modified Eagle's medium (DMEM), supplemented with 10% fetal bovine serum (Invitrogen, Carlsbad, CA, USA) at 37°C with 5% CO_2_.

### Survival rates of PEDV and PRV based on the TCID_50_ determination

Vero and PK-15 cells were cultured to be a monolayer, and then 96-wells were prepared by seeding with 7 × 10^4^ in each well. Viruses (PEDV and PRV) were performed a series of 1:10 of the original virus samples. The dilution samples of viruses (1 × 10^−6^) were obtained and the Piscidin 1 and lactoferricin concentrations were obtained with two-fold serial dilutions method by the virus dilution in the 96-well. Finally, the compound samples including cells, viruses and Piscidin 1 and lactoferricin were cultured at 37°C with 5% CO_2_. The absence of virus infectivity was confirmed and regarded as (A_1_) by 50 percent tissue culture infective dose with OD values determination. In addition, the viruses PEDV and PRV were incubated with 50 μM Piscidin 1 for 1 h, and the TCID50 also determined with the above operation and regarded as A_2_. The survival viruses rates of PEDV and PRV were calculated with the following as: R (survival viruses rates) = A_2_/A_1_ × 100%. Moreover, the statistically significant differences of R between Piscidin 1 and lactoferricin against PEDV and PRV were compared with Prism software.

### Plaque assay for piscidin 1 against PEDV and PRV

To determine the amount of infectious viruses (PEDV and PRV) under the different Piscidin 1 concentrations (0, 1, 2, 5, 10, 25 μg/ml), the plaque assay was conducted in this study. Ten dilutions of the viruses stock were prepared, and 0.1 ml aliquots were inoculated onto susceptible cell monolayers (Vero and PK-15 cells). After 1 h viral adsorption, the cells (Vero and PK-15) were washed with Dulbecco's modified Eagle medium (DMEM, Gibco, USA) and overlaid with sodium carboxymethyl cellulose including medium supplemented with trypsin or 2% FBS for PEDV or PRV, respectively. Finally, the plaques samples were fixed with 10% formaldehyde 3 days after infection and then stained with crystal violet solution (Ye et al., 2015; Deng et al., 2016).

## Comparative PK study for piscidin 1

### Animals

96 healthy, equal male and female specific pathogen-free Wistar rats (200–220 g) were procured from the Center of Laboratory Animals of Hubei Province (Wuhan, China). Among these Wistar rats, the 24 rats with male and female were selected for bioavailability study for Piscidin 1, the others were conducted for the PK profiles in intestinal tract after intramuscular injection (i.m) at 2 mg/kg administration.

All of the animals did not receive any antimicrobial treatment 14 days before the experiments. These animals were deemed to be normal and clinically healthy after having a regular body checkup and were thus used for this experiment. The basic feed of mice who did not undergo any drug administration was according to the Chinese standard “Laboratory animal rats and mice feed” (Parkes, 1946).

All experimental procedures performed with piglets, rats and monkeys have been approved by the Ethical Committee of the Faculty of Veterinary Medicine at Huazhong Agricultural University. All animal care and experimental protocols in the piglets, rats and monkeys were conducted in accordance with the Guide for the Care and Use of Laboratory Animals of Hubei Provincial Laboratory Animal Public Service Center (permit number SYXK 2013-0044).

### Experimental design

24 Wistar rats (50% females) were selected and randomly assigned to three groups (8 rats per group) A, B, and C. In these groups, A, B, and C were received a single dosage of 2 mg/kg of Piscidin 1 with intravenous injection (i.v), i.m, and per-os (p.o) administrations, respectively. Blood (1.2 ml) samples for three groups were collected at 0, 5, 10, 20, 30 min, 1, 2, 4, 8, 12, 24, 36, and 48 h by blooding the posterior orbital veins of rats.

The other 72 Wistar rats were also randomly divided into 12 groups (6 per group). All of the rats were i.m administrated with a single dose of 2 mg/kg Piscidin 1. Ileum content (1 g) was collected at the following time points after i.m administration of Piscidin 1:0, 5, 10, 20, 30 min, 1, 2, 4, 8, 12, 24, and 48 h.

### Blood and ileum content treatment

Blood samples with the anticoagulant were centrifuged at 3,000 rpm for 10 min, and then the serum samples were obtained. The 0.5 ml acetonitrile containing 5% acetic acid was added to the 200 μl serum in the tubes, then vortexed for 2 min and centrifuged at 12,000 rpm for 10 min. The aqueous phase was transferred into a clean tube and dried under nitrogen in a thermostat water bath at 50°C. The initial flow phase (200 μl) was used to dissolve the sample. The final samples were filtered through membrane filters with a pore size (0.22 μm) and analyzed using high-performance liquid chromatography-mass spectrometry (LC-MS/MS).

0.2 g ileum content sample was homogenized for 1 min, and 2 ml methanol containing 0.5% acetic acid was added to the homogenate samples. Then the mixture was vortexed for 2 min and centrifuged at 12,000 rpm for 10 min. The aqueous phase was transferred into a clean tube and dried under nitrogen in a thermostat water bath at 50°C. The initial flow phase (200 μl) was used to dissolve the sample. The final samples were filtered through membrane filters with a pore size (0.22 μm) and analyzed using high-performance liquid chromatography-mass spectrometry (LC-MS/MS).

### LC-MS/MS conditions for piscidin 1

The Piscidin-1 concentrations were measured by LC-MS which consists of an Ekspert Ultralc 100 apparatus (Eksigent, Redwood City, CA, USA) with a 5 μm Kinetex C_18_ column (50 mm × 2.0 mm, 5 μm, i.d., Phenomenex, Guangzhou, China) coupled to an AB sciex (Applied Biosystem/MDS SCIEX, Foster City, CA, USA) triple quad 5500 mass spectrometer equipped with an electrospray ionization source. The mobile phases were composed of a mixture of 0.5% formic acid in water (A) and 0.5% formic acid in methanol (B). The gradient elution used method was conducted from 30% B for 0.2 min, increased to 100% B within 0.8 min, held for 1.3 min, and decreased to 30% for 2.7 min. The flow rate was 0.4 ml/min, and the column temperature was kept at 35°C. To determine the Piscidin-1 concentration, the negative ion mode was selected, along with the followed conditions: spray voltage was set as 4.5 kV, sheath gas and auxiliary gas were 45 and 10 L/min, respectively, the capillary temperature was 300°C. Detection and quantification of Piscidin-1 were performed using multiple reactions monitoring (MRM) mode with monitoring of the precursor to product transitions.

### Method validation and PK analysis

The standard external method was performed for the determination of Piscidin-1 in serum and ileum content. The standard curve of Piscidin-1 from 0.02 to 10 μg/ml was detected by LC-MS/MS system both in plasma and ileum content. The linear regression, coefficient variation and recovery were also calculated.

The analysis of the concentration-time profiles and PK parameters of Piscidin-1 in serum and ileum content were performed using WinNonlin (version 5.2.1, Pharsight Corporation, Mountain View, CA, USA). The concentrations in plasma and ileum content were analyzed using non-compartmental model methods.

### Statistical analysis

The statistical analyses were conducted by the Student's *t*-test with using Prism software (Graphpad Software Inc., London, United Kingdom). *P*-values < 0.05 were considered to demonstrate statistically significant differences. Otherwise, it would indicate non-statistically significant differences.

## Results

### TCID_50_ determination of piscidin 1 and lactoferricin against PEDV and PRV

**Figure 1:**
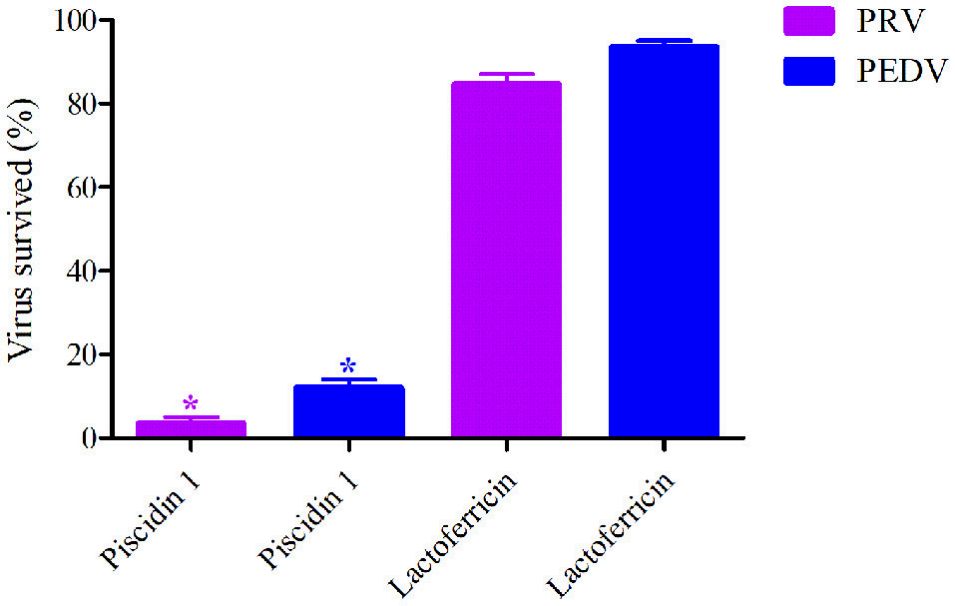
The survived virus ratios analysis after treatment by Piscidin 1 and Lactoferricin (50 μg/ml). *Means the statistically significant differences (*P* < 0.05).

Two kinds of peptides were selected for TCID_50_ determination against PEDV and PRV. The means of virus survived ratios of PRV and PEDV were shown to be 2%, 82%, and 12%, 93%, respectively after exposed to 50 μg/ml of Piscidin-1 and lactoferricin in Figure 1. Obviously, the virus survived ratios of PRV and PEDV after treatment by Piscidin-1 were significantly lower than those after treatment by lactoferricin, and it revealed statistically significant differences between each other (*P* < 0.05). This result demonstrated Piscidin-1 might have better effects against PEDV and PRV.

### Plaque reduction assay

**Figure 2:**
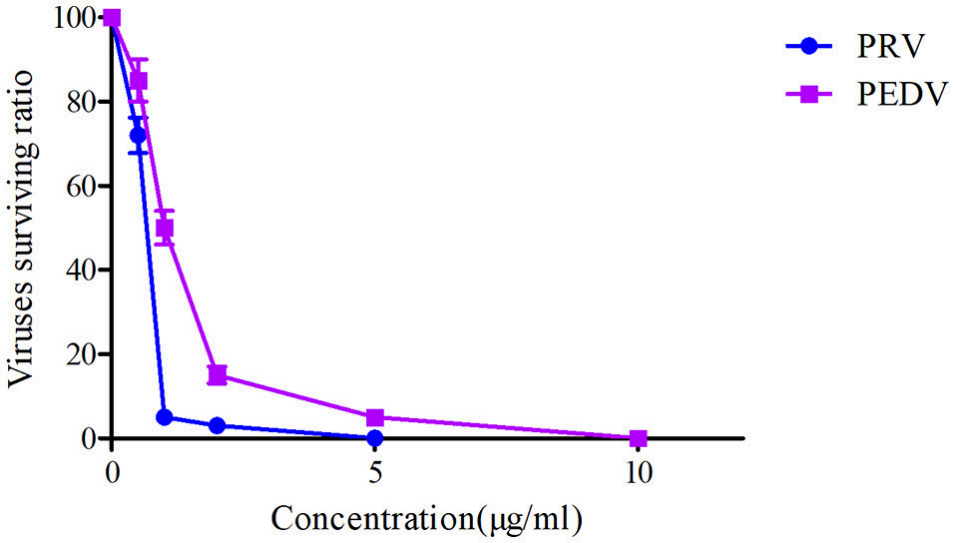
The viruses surviving ratio of PRV and PEDV based on the plaque reduction assay.

The plaque reduction assay for PRV and PEDV were performed after exposure to a serial of Piscidin-1 concentrations (0, 0.5, 1, 2, 5, 10, 25 μg/ml). The plaque test could be observed in Figure 2 and presented that 10 μg/ml of Piscidin-1 could restrain the growth of plaque belongs to PRV and PEDV. In addition, 1 and 2 μg/ml Piscidin-1 could significantly eliminate 95 and 85% plaque forming for PRV and PEDV, respectively (Figure 2). Five and ten microgram per milliliter could completely inhibit the plaque forming for PRV and PEDV, respectively in Figure 2. Furthermore, the result also indicated the inhibition of Piscidin-1 against PRV might be better than PEDV.

### Mass spectrometry

**Figure 3:**
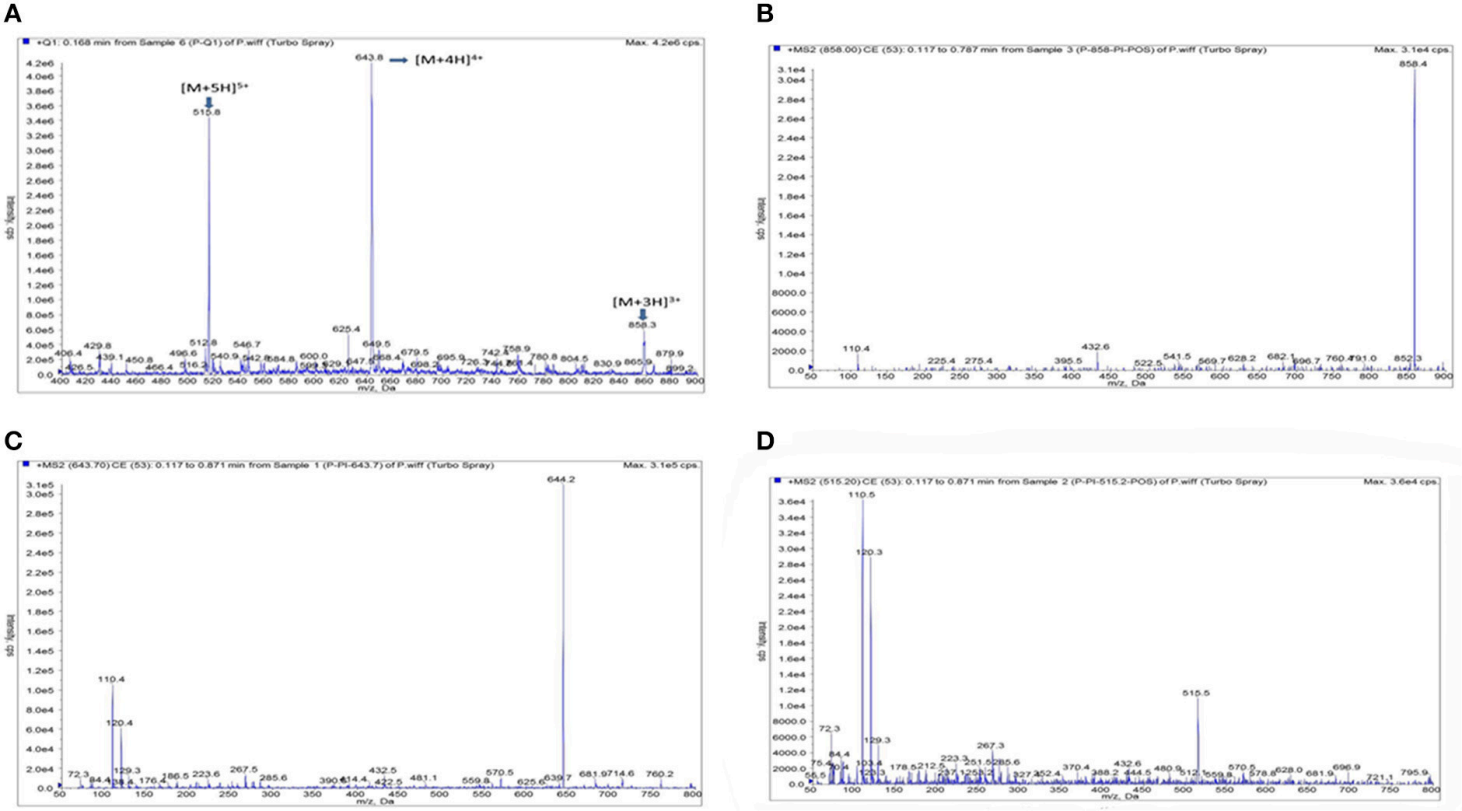
The product ion plots for Piscidin 1. **(A)** Means the full- full –scan mass spectrum analysis, **(B)** means the tri-charged precursor ion scans, **(C)** means the four-charged precursor ion scans, **(D)** means the five-charged precursor ion scans.

Based on the MS operating conditions in the method section, Piscidin-1 could be easily ionized to form precursor ions of [M+3H]^−^, [M+4H]^−^, and [M+5H]^−^ at m/z 858.4, 644.2, and 515.5.5 in negative ionization mode. The full-scan mass spectrum analysis was shown in the Figure 3A, the tri-, four-, and five-charged precursor ion scans were shown in the Figures 3B–D, respectively. It is obviously presented the ion transition of m/z 2575 → 644.2 which had the highest signal response, lowest noise and greater stability than others was selected for quantification of Piscidin-1.

### Validation of the LC-MS/MS method for piscidin 1

**Figure 4:**
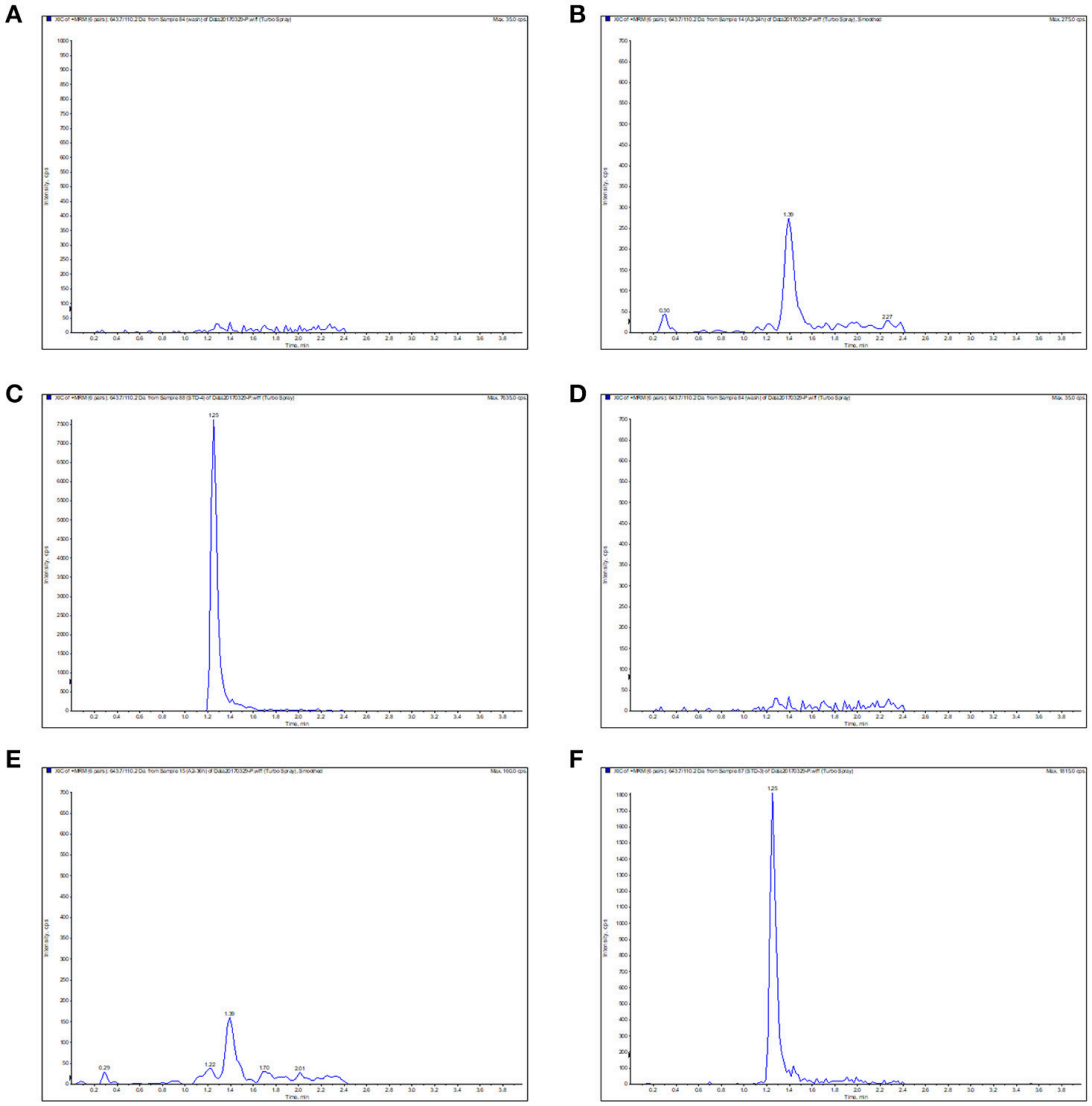
The typical LC-MS chromatograms for Piscidin 1 in the serum and ileum content. **(A)** Represents the blank serum sample, **(B)** represents the LOQ (0.02 μg/ml) in the serum, **(C)** represents the serum sample in 10 min, **(D)** represents the blank ileum content, **(E)** represents the LOQ (0.02 μg/g) in the ileum content, **(F)** represents the ileum content sample in 2 h.

The calibration curves presented a high linearity for Piscidin-1 detection ranged from 0.02 to 10 μg/ml or μg/g in both serum and ileum content. The correlation coefficients (R^2^) were calculated as 0.9976 and 0.9954 in the serum and ileum content, respectively. The lower limit of quantification (LOQ) and detection (LOD) were both 0.02 and 0.01 μg/ml or μg/g in both serum and ileum content. The blank sample, LOQ, samples were presented in Figure 4 for serum and ileum content, respectively. The inter- and intra-day variations were calculated to be 1.5–5% in the serum and ileum content. The recovery ratios were calculated in the range of 85–90% and 82–88% in the serum and ileum content, respectively. In a word, these result suggested the method of detecting Piscidin-1 in the serum and ileum content by LC-MS/MS met the requirements in bio-analytical domain.

### PK analysis of piscidin 1 after I.V, I.M and P.O administrations in the plasma

**Figure 5:**
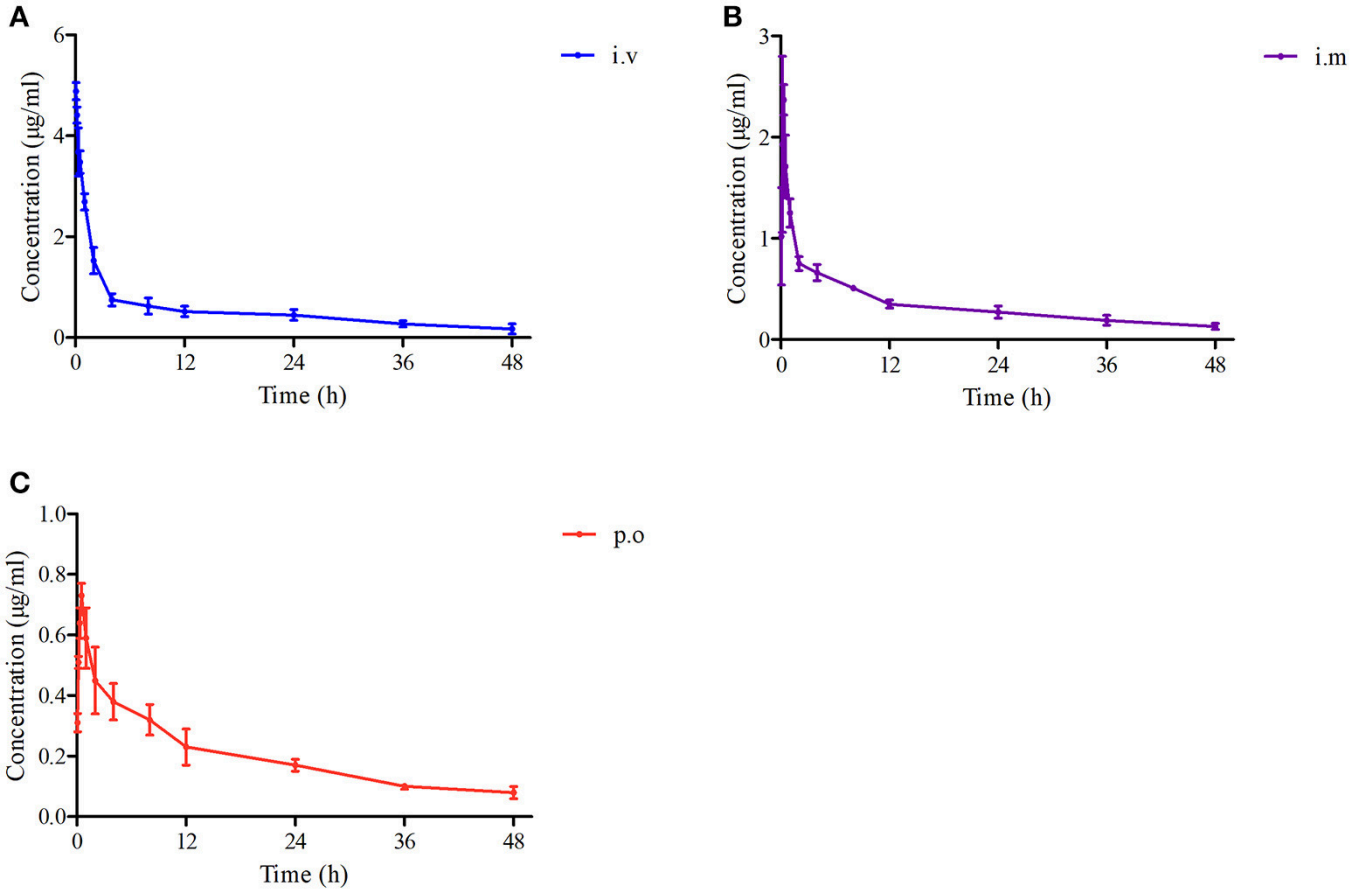
The drug concentration-time curves of Piscidin 1 after i.v, i.m and p.o administration (2 mg/kg). **(A)** Represents the curve after i.v administration, **(B)** represents the curve after i.m administration, **(C)** represents the curve after p.o administration.

The proposed methods of LC-MS/MS were appropriate for Piscidin-1 determination in the plasma and ileum content. The mean ± SD of Piscidin-1 concentrations-time profiles after i.v, i.m ,and p.o administrations were presented in the Figures 5A–C. The main PK parameters were shown in Table 1 with using non-compartment model. Moreover, the bioavailability (F) values for i.m and p.o administrations were calculated to be 73.17% and 37.18. These results revealed the concentrations of Piscidin-1 after i.m administration were higher than p.o administration and the route of i.m administration had more benefit for Piscidin-1 absorption. Therefore, it is recommended that the i.m administration for Piscidin-1 might the best route for the PK investigation in ileum content and veterinary clinic.

**Table 1:** The PK parameters (Mean ± SD) of Piscidin 1 in the rat via different administration.

**Parameters**	**Mean** ± **SD**		
	**i.v**	**i.m**	**p.o**
AUC_0−∞_(μg/ml*h)	25.90 ± 3.21	18.95 ± 1.37*	9.63 ± 0.87
MRT(h)	22.77 ± 2.43	30.12 ± 2.46	26.76 ± 2.89
Ke(h^−1^)	0.041 ± 0.009	0.029 ± 0.006	0.036 ± 0.004
T_max_(h)	–	0.333 ± 0.151	0.50 ± 0.23
CL_b_ (L/h/kg)	0.067 ± 0.02	0.095 ± 0.01*	0.171 ± 0.08
T_1/2_(h)	16.97 ± 1.43	23.50 ± 2.43	19.46 ± 1.67
C_max_(μg/ml)	–	2.37 ± 0.16*	0.73 ± 0.08
F		73.17%	37.18%

* Means the statistically significant differences (p < 0.05).

*Ke, presents elimination rate constant, T_1/2_, presents elimination half-life, T_max_, the time point to reach the peak concentration, Cl_b_, presents body clearance, AUC_0−∞_, presents area under the curve, C_max_, peak concentration, MRT, presents mean retention time*.

### PK analysis of piscidin 1 after I.M administrations in the ileum content

**Figure 6:**
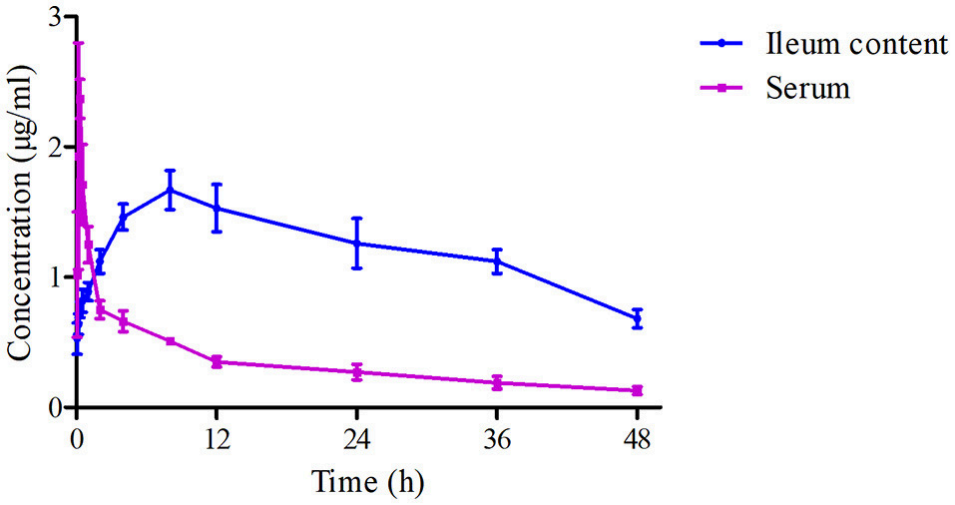
The drug concentration-time curves of Piscidin 1 in the serum and ileum content after i.m administration (2 mg/kg).

The mean ± SD of Piscidin-1 concentrations-time profiles after i.m administration was presented in Figure 6. The main PK parameters for Piscidin-1 in the serum and ileum content were shown in Table 2 with using non-compartment model. The AUC_0−∞_, MRT, Ke, T_max_, CL_b_, T_1/2_, and C_max_ were calculated as 78.40 ± 8.64 μg/g^*^h, 36.45 ± 2.45 h, 0.034 ± 0.06 h^−1^, 8.12 ± 1.02 h, 0.026 ± 0.008 L/h/kg, 20.16 ± 1.76 h and 1.67 ± 0.19 μg/g, respectively. The parameters in the serum had been shown in Table 1, and these were also compared to those in ileum content. There were statistically significant differences for AUC_0−∞_, T_max_, and CL_b_ between in the ileum content and serum (*P* < 0.05). The means of AUC_0−∞_ and T_max_ were over 4 and 24 times higher in the ileum content than serum. The mean of CL_b_ was 0.273 times lower in the ileum content than serum and the elimination constants (K_e_) were practically equal (0.29 and 0.34), the differences in clearance were the consequence of differences of volume of blood and volume of ileum content. Actually, the parameters in the ileum content would reveal the authentic and immediate drug concentrations, and the ileum content was the site where were the viruses (PEDV) infecting and cloning. These results might have more practical significance for a veterinary clinic.

**Table 2:** The PK parameters (Mean ± SD) of Piscidin 1 in ileum content after i.m administration (2 mg/kg).

**Parameters**	**Ileum content**	**Serum**	**Ratio**
AUC_0−∞_(μg/ml*h or μg/g*h)	78.40 ± 8.64*	18.95 ± 1.37	4.14
MRT(h)	36.45 ± 2.45	30.12 ± 2.46	1.21
Ke (h^−1^)	0.034 ± 0.06	0.029 ± 0.006	1.17
T_max_(h)	8.12 ± 1.02*	0.333 ± 0.15	24.38
CL_b_ (L/h/kg)	0.026 ± 0.008*	0.095 ± 0.01	0.273
T_1/2_(h)	20.16 ± 1.76	23.50 ± 2.43	0.858
C_max_(μg/ml or μg/g)	1.67 ± 0.19*	2.373 ± 0.16	0.71

* Means the statistically significant differences (p < 0.05).

*Ke, presents elimination rate constant, T_1/2_, presents elimination half-life, T_max_, the time point to reach the peak concentration, Cl_b_, presents body clearance, AUC_0−∞_, presents area under the curve, C_max_, peak concentration, MRT, presents mean retention time*.

## Discussions

To date, there were rare reports on the AMPs against viruses and bacteria, and the PK profiles of AMPs were also lack of investigation especially in the target infection tissues. In the current study, Piscidin-1, which belonged to piscidin families, was first selected for the preliminary study of the PD on PEDV and PRV, based on the determination of TCID_50_ and plaque reduction assay, and the characteristics PK of plasma tissue and PEDV target infection tissue (ileum content) after three administrations (i.v, i.m, and p.o) at a dose rate of 2 mg/kg. As a new type of therapeutic agent, AMPs such as Piscidin-1 are not only developed for their antimicrobial effect, but also play a role in the innate and adaptive immune response to viruses that would be developed as potential novel agents instead of antibiotics in the future (Lee et al., 2007; Yuan et al., 2012; Lin et al., 2016; Kumar et al., 2017).

Generally, the antimicrobial activity of AMPs (Capp 18, Cap 11, Cecropin P1, Cecropin B, Piscidin 1, and so on) against a range of pathogenic bacteria (*S. aureus, Enterococcus faecalis, P. aeruginosa, E. coli, Aeromonas salmonicida, Listeria monocytogenes, Salmonella typhimurium, Campylobacter jejuni, Flavobacterium psychrophilum*, and *Yersinia ruckeri*) had been investigated with MIC determinations in the previous reports (DoleŽílková et al., 2011; Moghaddam et al., 2012; Ebbensgaard et al., 2015). These previously published reports revealed that Cap 18 belong to those AMPs had the highest activity with MICs ranged from 1 to 8 μg/ml to the most tested bacteria (excluding the *Flavobacterium psychrophilum* and *S. aureus*, whose MICs were determined as over 32 μg/ml). Furthermore, Piscidin-1 a kind of novel and potential AMP, belong to the piscidin family, the MICs of it against various bacteria had also been reported, and it had been shown that Piscidin-1 was the optimal AMP among the piscidin families. The MICs of Piscidin-1 against *E. coli, P. aeruginosa, S. aureus*, and so on were detected and ranged from 2.5 to 5 μg/ml which were the lowest values compared to other piscidin families in the previously published study (Colorni et al., 2008; Noga et al., 2009; Park et al., 2011; Moghaddam et al., 2012; Lee et al., 2014; Ebbensgaard et al., 2015; Jeong et al., 2016). Those results could demonstrate that Piscidin-1 had the outstanding profiles either against G^+^ or G^−^ adequately, and might be the more potent for the development of antibiotic alternatives. Generally, Piscidin-1 as other AMPs was developed for antibiotic alternatives. In this study, Piscidin-1 was firstly selected to investigate the antiviral activity of PEDV and PRV.

In the current study, another antimicrobial peptide (lactoferricin) was selected to investigate the antiviral activity for PEDV and PRV compared with Piscidin-1. The results of these two peptides against PEDV and PRV demonstrated that the virus survived ratios of PRV and PEDV after treatment by Piscidin-1 were presented significantly lower than those after treatment by lactoferricin (*P* < 0.05). In other words, the antiviral activity of Piscidin-1 was significantly higher than lactoferricin for these two viruses. Due to the bio-specificity of different antimicrobial peptides, the antiviral activity of the various peptides to the particular virus could present the diversities. In this study, the surviving ratios of PRV and PEDV after treatment with Piscidin-1 were 2 and 12%, respectively, indicating that Piscidin-1 had an excellent character at PRV and PEDV. The results were also the first to study the relationship for Piscidin-1 to PRV and PEDV. The plaque test presented that 10 μg/ml of Piscidin-1 could restrain the growth of plaque belongs to PRV and PEDV. In addition, 1 and 2 μg/ml Piscidin-1 could significantly eliminate 95 and 85% plaque forming for PRV and PEDV, respectively (Figure 2). Five and ten Microgram per milliliter could completely inhibit the plaque forming for PRV and PEDV, respectively in Figure 2. These result also indicated that Piscidin-1 had an excellent profile against PRV and PEDV.

As a novel and potential substitute for antibiotics, AMPs has attracted more and more attention in the field of veterinary medicine and human medicine. The *in vitro* antimicrobial activities of Piscidin-1 and its analogs (L or D-lysine residues) against a variety of bacteria had been reported in the previously published studies by Lee and Kumar (Lee et al., 2014; Kumar et al., 2017). However, some researchers drew attention to *in vivo* PK study of AMPs such as Piscidin-1 in target or model animals. The reasons could be the AMPs were hard to obtain, and produced with chemical synthesis mostly (Wang et al., 2014). To our knowledge, this is the first report of the PK study containing the three different routes of administrations (i.v, i.m, and p.o) and comparison of the bioequivalence of Piscidin-1 in Wistar rats. In the current study, Piscidin-1 was administrated with three routes (i.v, i.m, and p.o) at a single dosage of 2 mg/kg, the PK data in the plasma for AUC_0−∞_, MRT, Ke, T_max_, CL_b_, T_1/2_, and C_max_ were shown in the Table 1 after i.v, i.m and p.o administrations, respectively. It is obviously observed that the PK parameters for AUC_0−∞_, MRT, T_1/2_, and C_max_ (18.95 μg/ml^*^h, 30.12 h, 23.50 h, 2.37 μg/ml) after treatment by i.m administration were higher than those (9.63 μg/ml^*^h, 26.76 h, 19.46 h, 0.73 μg/ml) by p.o administration. The means of AUC_0−∞_ and C_max_ administrated by i.m were significantly higher than those by p.o (*P* < 0.05). These results meant that Piscidin-1 after treatment with the i.m administration had higher plasma concentrations of drugs, longer residence time, and slower elimination rate than that treated by the p.o administration. Furthermore, the PK parameters of Ke, T_max_, and CL_b_ treated by i.m administration were lower than those by p.o administration. Particularly, the value of CL_b_ treated by i.m administration had a significant difference (*P* < 0.05) compared to that by p.o administration. These parameters results revealed that Piscidin-1 had presented a rapid absorption, but slow elimination *in vivo* (plasma) after i.m administration. F% after i.m administration (73.17%) was 2 times higher than that after p.o administration (37.18%). This demonstrated that the i.m route of administration would be the best drug delivery scheme for Piscidin-1. Moreover, the lower bioavailability from p.o administration for Piscidin-1 might be attributed to the enzymolysis by animals, since Piscidin-1 was a kind of polypeptide belongs to the protein. It was easy to be hydrolyzed by a variety of enzymes in the oral cavity and gastrointestinal tract. This viewpoint had been reported in the published studies (Jiang et al., 2014; Chen et al., 2015; Zhou et al., 2017). Therefore, it has been suggested that Piscidin-1 be better administered by the i.m or i.v administration for the veterinary practice in this study.

As we know, PEDV is the serious virus disease, infecting intestinal tract in pigs. Thus, it is essential to investigate the PK profiles with an appropriate route in ileum content for Piscidin-1. In the current study, the drug concentrations (Piscidin-1) and retention time in the target infection tissues (ileum content) were significantly higher and longer than those in the serum which could be observed in Table 2. There had been no any other studies on Piscidin-1 in the ileum content, and this study was the first time to evaluate the PK profiles for Piscidin-1. The parameters of AUC_0−∞_, Ke, T_max_, and CL_b_ for Piscidin-1 in the ileum content after i.m administration had a significant difference (*P* < 0.05) compared to those after p.o administration. After oral administration a part of Piscidin-1 was absorbed and a part remains in intestine so that this could be an explanation for the fact that concentration in plasma was lower than concentration after i.m or i.v administration. All these facts were compatible with a bio-compartmental pharmacokinetic model of Piscidin-1, the compartments being plasma and ileum content. This difference for CL_b_ of Piscidin-1 in ileum content and serum revealed that it might have the strong penetration ability for various tissues and easily accumulated in intestinal contents after i.m administration. Moreover, the higher AUC_0−∞_ and T_max_ (78.40 μg/g^*^h, 8.12 h) devised from ileum content demonstrated that Piscidin-1 could penetrate membranes and tissues, binding to the solid parts of the ileum content which could cause the therapeutic action on the bacteria or viruses (Ahmad et al., 2015; Sang et al., 2016; Wang et al., 2016). According to the drug concentration-time curves and PK parameters in the ileum content and serum after i.m administration (2 mg/kg), the C_max_ values in plasma (2.37 μg/ml), and ileum content (1.67 μg/ml) were higher than the plaque test results and could restrain 85% propagation for PEDV. In addition, drug concentrations in the contents of the ileum may remain high and stabilized, indicating that Piscidin-1 may play a continuous role against PEDV in the ileum content. However, it is still necessary to establish a more adequate scheme in the subsequent study, since concentrations in the ileum or serum content cannot reach the value of more than 5 μg/ml and eliminate the PEDV absolutely after i.m administered dosage (2 mg/kg) of Piscidin-1. A more dosage scheme for Piscidin-1 would be better to cure the PEDV, which should be validated and clinically applied in future studies.

## Conclusion

AMPs as a new type of therapeutic agent are not only developed for their antimicrobial effect, but also play a role in the innate and adaptive immune response against viruses. It had been demonstrated that 1 and 2 μg/ml Piscidin-1 could significantly eliminate 95 and 85% plaque forming for PRV and PEDV, respectively. Piscidin 1 could show a strong inhibiting effect against PRV and PEDV *in vitro*. In addition, it had been recommended i.m administration could be a better route with a higher bioavailability (73.17%) for Piscidin-1 in Wistar rats, and the PK characteristics in the target tissues (ileum content) had presented the concentrations (Piscidin-1) could be kept higher, more stabilized, and eliminated more slowly *in vivo*. These demonstrated that Piscidin-1 might have good effect against PEDV *in vivo* and could be regarded as an alternative antibiotic in clinical veterinary in the future study.

## Author contributions

JC and QL conceived this study. QL and ZL designed the experiments. ZL, AS, QZ, YS and BY performed the experiments. ZL wrote the manuscript. QH, HK, YQ, JW, GM, and JC improved the language. All authors reviewed the manuscript.

### Conflict of interest statement

The authors declare that the research was conducted in the absence of any commercial or financial relationships that could be construed as a potential conflict of interest.

## Abbreviations

AMPS: antimicrobial peptides
MIC: minimum inhibitory concentration
PEDV: porcine epidemic diarrhea virus
PRV: pseudorabies virus
TCID_50_: 50 percent tissue culture infective dose
LLOD: limit of detection
LLOQ: limit of quantitation
K_e_: elimination rate of constant
t_1/2_: half-life of elimination
Cl_b_: the total body clearance
MRT: mean residence time
AUC_0−∞_: area under curve from 0 to ∞
T_max_: time to the concentration peak
C_max_: the concentration in the peak
F: biovailability
PD: pharmacodynamics
PK: pharmacokinetics
LC-MS/MS: high-performance liquid chromatography-mass spectrometry.
